# Effects of aspect ratio and metal layer thickness on demoulding of metal/polymer bilayer gratings during nanoimprinting

**DOI:** 10.1038/s41598-018-31194-y

**Published:** 2018-08-24

**Authors:** Xu Zheng, Qing Wang, Rui Zhang, Lijun Ma, Jinjin Luan

**Affiliations:** 1 School of Civil Engineering and Architecture, Shandong University of Science and Technology, Qingdao, Shandong 266590 China; 2 Institue of NanoEngineering, Shandong University of Science and Technology, Qingdao, Shandong 266590 China

## Abstract

During the fabrication of metal/polymer bilayer gratings by nanoimprint lithography, adhesion and friction forces at the interfaces can deform and damage the transferred pattern of the bilayer grating during the demoulding process. To improve the quality of bilayer gratings, the effects of the aspect ratio and metal layer thickness on deformation and stress during the demoulding process in the nanoimprinting of bilayer gratings were investigated. This information was used to determine the optimal grating parameters. The results for the von Mises stress and deformation of bilayer gratings are discussed in detail. The effects of the aspect ratio and metal layer thickness on the grating quality are then considered.

## Introduction

Metal gratings are useful in applications such as flexible sensors, solar cells, batteries, reflective polarizers, and displays^1–4^, so fabricating metal gratings is an important issue. Fine metal grating structures were initially fabricated by direct nanoimprint lithography (NIL) at ultra-high pressure and high temperature^5–7^. The direct NIL of metal gratings is possible in some cases, but the ultra-high pressure and high temperature conditions can damage the underlying substrate or device^8^. This limitation promoted the development of a method to fabricate metal/polymer bilayer grating structures^9,10^. This method greatly lowers the imprinting pressure and temperature compared with those needed for direct NIL.

The NIL of metal/polymer bilayer structures has been extensively studied both experimentally and numerically, in attempt to improve the method. Chen *et al*. patterned metal/polymer bilayer structures by NIL with a sharp-tip mould, which lowered the required imprinting pressure^9^. Chen and Nagpal fabricated various metal/polymer bilayer two-dimensional and three-dimensional structures by NIL^10,11^. Mori and Matubayasi explored the filling of epoxy resin in a nanosized pore in an aluminium surface. They aimed to better understand problems associated with the filling process, by using all-atom molecular dynamics simulations^12^. The performance of a grating depends on its particular application and thus its design. Sung and Maria explored the effects of the period and depth during the filling process on the performance of bilayer sensors^2,13^. The period of the grating and thickness of the metal layer were found to greatly influence the transmittance. Duempelmann and Zheng fabricated and characterized a large-area bilayer grating^14,15^. Distinctive colours spanning the entire visible spectrum could be generated by tuning the system parameters, such as the period and length of the aluminium structure. The grating parameters such as the period, aspect ratio, and height therefore greatly affected its optical performance.

To analyse the effects of the mould geometry and imprinting process parameters on the quality of pattern transfer in a direct nanoimprint process, the formation height ratio and imprint pressure during imprinting were evaluated experimentally and theoretically using the finite element method (FEM)^16,17^. Many studies have investigated the bilayer structure filling process and the effects of different shapes. However, the demoulding of bilayer structures has seldom been studied. This is despite the demoulding process playing an important role in determining the quality and performance of the nanostructure^18^.

In the current study, two major factors causing pattern destruction in the demoulding process, i.e., the aspect ratio and metal layer thickness, are investigated. The maximum stress and deformation of bilayer gratings during the demoulding process are studied by FEM simulation of these two factors. The simulation results reveal the variation of the maximum stress and deformation with varying aspect ratio and metal layer thickness. The simulation model and numerical results provide an understanding of the mechanism of the demoulding process, and can be used to estimate the optimal grating parameters.

## Results and Discussion

**Figure 1:**
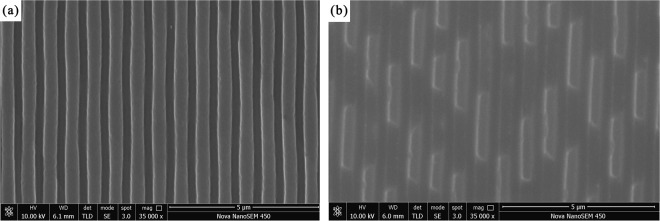
SEM images of the grating of (**a**) the mold and (**b**) the metal/polymer bilayer.

Figure 1(a,b) show scanning electron microscopy (SEM) images of the grating of the mould and metal/polymer bilayer, respectively, obtained using a scanning electron microscope (Nova Nano SEM450, FEI, USA). The grating mould has a period of 700 nm and an aspect ratio of 1.5. The metal/polymer bilayer grating was fabricated by the method described below using the grating mould. Figure 1(b) reveals that the bilayer grating pattern is damaged. The angle of the grating is a right angle. This indicates that the pattern has a high filling rate, and that the defects occur in the demoulding process. Most of the bilayer grating has been pulled off at the bottom, and part of the metal layer is damaged. This result suggests that the aspect ratio and thickness of the metal layer are important factors affecting the success of the demoulding process. Therefore, the aspect ratio and thickness of the metal layer were explored using FEM simulations.

**Figure 2:**
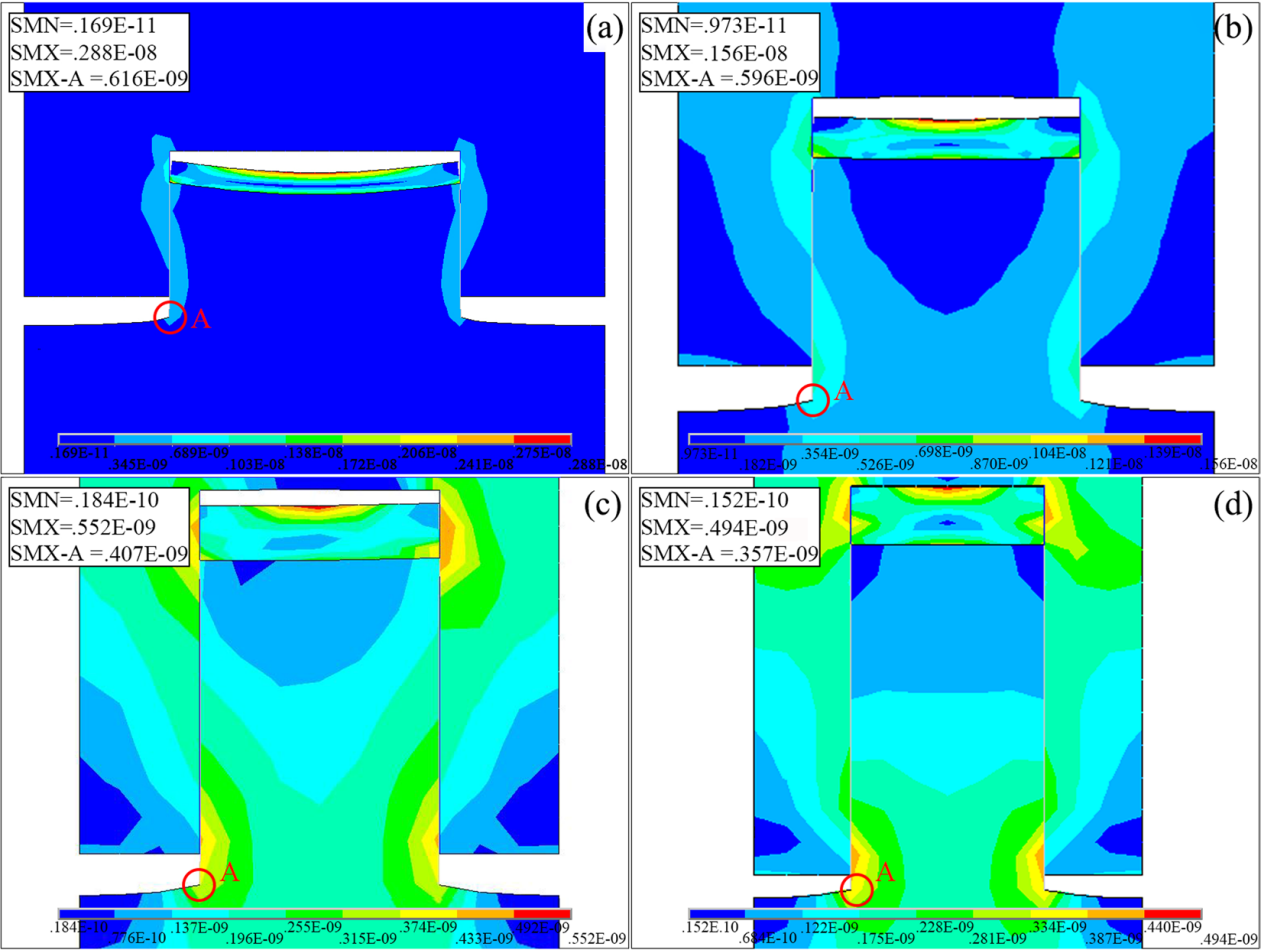
Von Mises stress distribution in bi-layer grating for metallic thickness of 30 nm with aspect ratio of (**a**) 0.5, (**b**) 1.0, (**c**) 1.5, (**d**) 2.0.

Figure 2 shows the von Mises stress distribution in the bilayer grating with a metal layer thickness of 30 nm for different aspect ratios during the demoulding process. The stress is concentrated at two different locations in the bilayer grating: the top centre of the metal layer and the bottom of the polymer layer (section A in Fig. 2). The stress at the top centre of the metal layer in Fig. 2(a–d) is 2880, 1560, 552, and 494 MPa, respectively. The stress at the bottom of the polymer layer in Fig. 2(a–d) is 616, 596, 407, and 357 MPa, respectively. Tensile deformation and migration occur at the bottom of the polymer layer during the demoulding process.

**Figure 3:**
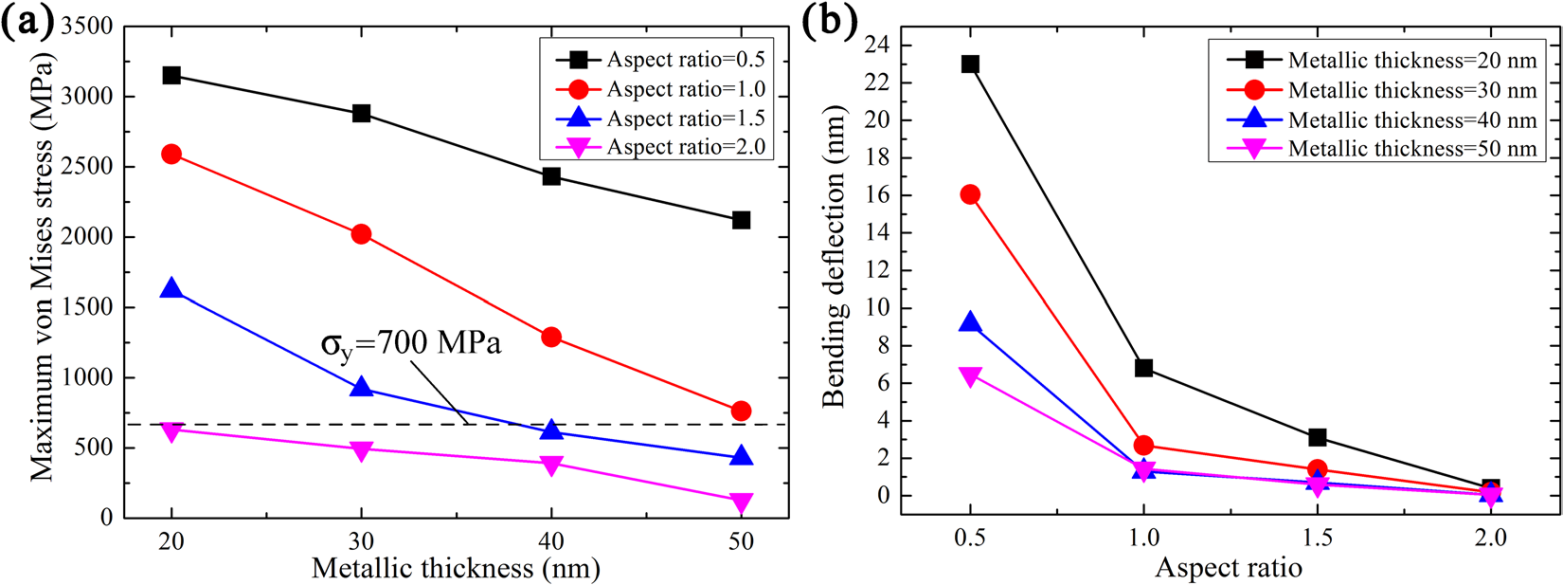
(**a**) Maximum von Mises stress and (**b**) bending deflection at the top center of the metal layer for gratings with various structural parameters.

To explore the effects of aspect ratio on the stress and deformation of metal/polymer bilayer gratings, various structural parameters were simulated by the FEM. Figure 3(a) illustrates the maximum von Mises stress at the top centre of the metal layer, for metal layer thicknesses ranging from 20 to 50 nm and for various aspect ratios. For a constant metal layer thickness, the maximum stress at the top centre of the metal layer increases as the aspect ratio decreases from 2.0 to 0.5. Previous studies reported that the stress was lowered by decreasing the aspect ratio in nanoimprinting, but only for polymers without a metal layer^19^. In the demoulding of a metal/polymer bilayer grating, there is a negative correlation between the maximum stress at the top centre of the metal layer and the aspect ratio.

During the demoulding process, the bending deformation of the metal layer can be determined from the contact force being applied by the mould. Figure 3(b) summarizes the bending deflections at the top centre of the metal layer for systems with various structural parameters. The bending deflection refers to the maximum relative vertical displacement between the top centre node and top corner node of the metal layer during demoulding. For a constant metal layer thickness, the bending deflection of the metal layer increases as the aspect ratio decreases from 2.0 to 0.5. Hooke’s law indicates that stress is proportional to the elasticity modulus under a constant strain,

1 $$\sigma =E{\varepsilon }{,}$$

where *σ*, *E*, and *ε* are the stress, elasticity modulus, and strain, respectively. A metal layer that shares the same nodes as the polymer layer will display the same deformation. The elasticity modulus of the metal layer (168 GPa) is much higher than that of the polymer layer (3.3 GPa). Thus, the stress of the metal layer is much higher than that of the polymer layer at the same deformation.

The metal layer thickness plays an important role in the demoulding process, especially regarding the resistance to bending deformation. Figure 3(a) reveals that for a constant aspect ratio, the maximum stress at the top centre of the metal layer decreases as the metal layer thickness increases from 20 to 50 nm. The maximum von Mises stress and metal layer thickness are negatively correlated. Bending deformation occurs in the metal layer during the demoulding process. As shown in Fig. 3(b), when the aspect ratio is 0.5 or 1.0, the bending deflection of the metal layer decreases as the metal layer thickness increases from 20 to 50 nm. When the aspect ratio is 1.5, the bending deflection still largely follows this trend. The bending deflection values are comparable for all metal thicknesses at an aspect ratio of 2.0. According to material mechanics, the flexural stiffness of a material determines its flexural performance. The section moment of inertia (*I*) is the critical factor for flexural stiffness when materials have the same elasticity modulus. The flexural stiffness and *I* are described by,

2 $${Flexural}\,{stiffness}={EI},$$

3 $$I=\frac{1}{{\rm{12}}}b{h}^{3},$$

where *b* and *h* are the section width and height, respectively. We found that *I*_*d*=50_ > *I*_*d*=40_ > *I*_*d*=30_ > *I*_*d*=20_ by calculating *I* for the various metal layer thicknesses. Thus, the bending deflection should decrease with increasing metal layer thickness. This is in good agreement with the results shown in Fig. 3(b).

As shown in Fig. 3(a), the maximum stress of the metal layer is below the yield strength for systems with an aspect ratio of 2.0 and metal layer thicknesses of 20 to 50 nm. The maximum stress of the metal layer is also below the yield strength for systems with an aspect ratio of 1.5 and metal layer thicknesses of 40 and 50 nm. This indicates that the metal layer only exhibits elastic deformation during the demoulding process under these conditions. When the metal thickness is 30 nm and the aspect ratio is 1.5, the maximum stress is greater than the yield strength. This is the reason for the defects forming in the experimentally prepared grating patterns. When the metal layer thickness is 50 nm and the aspect ratio is 1.0, the maximum stress of the metal layer is 763 MPa, which is close to the yield strength. As shown in Fig. 3(b), the deflection deformation is very small (1.4 nm) in this case. For the systems with the other structural parameters, the maximum stress is much higher than the yield strength. These conditions with high stress result in damage to the bilayer grating. Therefore, the seven conditions described above are suitable for use in the demoulding process, from the viewpoint of the maximum stress of the metal layer.

**Figure 4:**
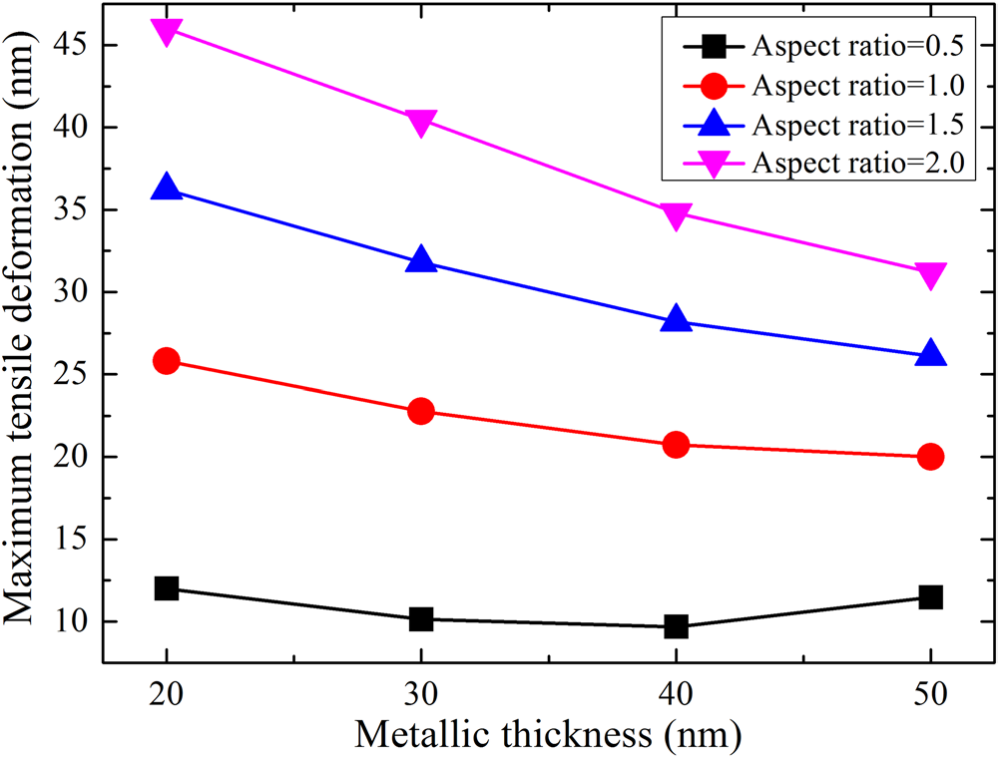
Maximum tensile deformation of the polymer layer for gratings with various structural parameters.

The polymer layer is an important component of bilayer gratings, so it is necessary to analyse the deformation of the polymer layer during demoulding. Figure 4 presents the maximum tensile deformation of the polymer layer for systems with various structural parameters. The maximum tensile deformation is the maximum vertical deformation at the top centre of the polymer layer during the demoulding process. As shown in Fig. 4, the tensile deformation of the polymer layer increases with increasing aspect ratio. For aspect ratios from 1.0 to 2.0, the tensile deformation of the polymer layer decreases as the metal layer thickness increases. When the aspect ratio is 0.5, the metal layer thickness has little effect on the tensile deformation of the polymer layer. The maximum tensile deformation is approximately 35 nm for an aspect ratio of 2.0, which is 17.5% of the mould depth. The maximum tensile deformation is approximately 30 nm for an aspect ratio of 1.5, which is 15% of the mould depth. In the practical experiments, the bilayer grating is easily damaged by such a large deformation. However, the maximum tensile deformation is approximately 20 nm for an aspect ratio of 1.0, and the deformation decreases by 43% and 33% compared with those for aspect ratios of 2.0 and 1.5, respectively. Therefore, an aspect ratio of 1.0 and metal layer thickness of 50 nm are suitable for use in the demoulding process, from the viewpoints of tensile deformation of the bilayer grating and maximum stress of the metal layer.

To conclude, the experimental results show that the angle of the grating is a right angle. This indicates that the pattern has a high filling rate, and that the defects occur in the demoulding process. Therefore, the effects of the aspect ratio and the metal layer thickness of bilayer gratings on their demoulding process in NIL were studied by FEM simulations. For a constant metal layer thickness, the maximum stress and bending deflection of the metal layer increase with decreasing aspect ratio. For a constant aspect ratio, the maximum stress and metal layer thickness are negatively correlated. For a constant metal layer thickness, the maximum tensile deformation of the polymer layer increases with increasing aspect ratio. Considering the maximum stress of the metal layer and maximum tensile deformation of the polymer layer, an aspect ratio of 1.0 and metal layer thickness of 50 nm are the most suitable for the demoulding process, among the considered cases. These findings will be useful for optimizing the experimental conditions in the NIL of bilayer gratings. The simulation could also be extended to explore the influences of the linewidth-to-pitch ratio and the optimized method on the demoulding outcome.

## Methods

### Experiment

Direct imprint technology was used to fabricate the metal/polymer bilayer grating. This process involved four steps: (a) The metal layer (Pt-Pd alloy, 30 nm thick) was deposited by vacuum vapor deposition technology (KYKY, VZZ-300, P. R. China) on the Ni mould surface; (b) Removal of the metal layer on the top of the embossed structure by chemical mechanical polishing; (c) Pressing of the mould with the metal layer into a polymethyl methacrylate (PMMA) layer by hot embossing imprinting (Imprint Nano, NIL-150, P. R. China) at 130 °C and 0.5 MPa for 10 min; (d) Separation of the cured mould from the resist by demoulding. And the surface energy of Ni, Pd and PMMA is 0.4 J/m^2^, 0.18 J/m^2^ and 21.6 MJ/m^2^, respectively^20,21^. Therefore, the adhesion of bilayer is much larger than that of metal layer/Ni interface, so that the metal layer can be transferred from the surface of the mould to the polymer surface by way of the surface energy difference.

### Simulations

To explore factors affecting the quality of the pattern obtained after demoulding, the demoulding process of the bilayer grating was investigated by FEM simulation using ANSYS software. As in the experiment, PMMA was used as the underlying polymer layer on the substrate. Then, a Pt-Pd layer was deposited on the mould. A Ni mould was used in this simulation. The anti-adhesion layer can effectively reduce the interface adhesion problem of mould and polymer. However, to understand the damage process of grating in the demoulding process more clearly, no anti-adhesion layer was added in the simulation. The PMMA was defined as a rubber elastic material, and the metal layer and mould were defined as hard elastic materials. The Mooney-Rivlin model was used to describe the PMMA resist layer^22,23^. In all simulations, the Young’s moduli of the Ni mould, Pt-Pd metal layer, and PMMA were 207, 168, and 3.3 GPa, respectively. The Poisson ratios of the Ni mould, Pt-Pd metal layer, and PMMA were 0.31, 0.39, and 0.15, respectively^24,25^.

To explore the demoulding process of nanoimprint lithography, the simulation conditions assumed that the bilayer grating completely filled the mould cavity in the original state. Regarding the boundary conditions, the bottom surface of the PMMA resist was fixed, and only the horizontal displacement on both sides of the model was confined. To complete the demoulding process, the interface between the Ni mould and bilayer grating was defined as slip-allowed. The mould was released by movement of a total distance of 200 nm in the upwards direction, which accomplished the demoulding process. In the simulation, the element PLANE 42 was used to represent the Ni mould and metal layer, and the element PLANE 182 was used to represent the PMMA resist. The contact element CONTA 171 was applied to the interface between the Ni mould and bilayer grating, representing the slip-allowed boundary. In addition, the interface between the metal and polymer layer shared the same nodes. The geometric parameters of the mould used in the simulation are listed in Table 1. The space and linewidth refer to the width of the groove and the width of the embossed region, respectively. To study the influences of the aspect ratio and metal layer thickness of the bilayer grating on demoulding, 16 cases with various structural parameters of the bilayer grating were simulated, as summarized in Table 1. The investigated aspect ratios (depth-to-space ratio) were 0.5, 1.0, 1.5, and 2.0. The linewidth-to-pitch ratio of the mould was constant (0.5). Metal layer thicknesses of 20, 30, 40, and 50 nm were investigated.

**Table 1:** Mold geometries used in simulations.

No.	Depth (nm)	Space (nm)	Linewidth (nm)	Pitch (nm)	Aspect Ratio	LPR
1	200	400	400	800	0.5	0.5
2	200	200	200	400	1.0	0.5
3	200	132	132	264	1.5	0.5
4	200	100	100	200	2.0	0.5
